# A 3D-printed magnetic digital microfluidic diagnostic platform for rapid colorimetric sensing of carbapenemase-producing *Enterobacteriaceae*

**DOI:** 10.1038/s41378-021-00276-9

**Published:** 2021-06-12

**Authors:** Pojchanun Kanitthamniyom, Pei Yun Hon, Aiwu Zhou, Mohammad Yazid Abdad, Zhi Yun Leow, Nurhidayah Binte Mohamed Yazid, Vanessa Lim Wei Xun, Shawn Vasoo, Yi Zhang

**Affiliations:** 1 Singapore Centre for 3D Printing, School of Mechanical and Aerospace Engineering, Nanyang Technological University, Singapore City, Singapore; 2 National Center for Infectious Disease, Tan Tock Seng Hospital, Singapore City, Singapore; 3 Lee Kong Chian School of Medicine, Nanyang Technological University, Singapore City, Singapore

**Keywords:** Engineering, Chemistry

## Abstract

Carbapenemase-producing *Enterobacteriaceae* (CPE) are a group of drug-resistant Gram-negative pathogens that are classified as a critical threat by the World Health Organization (WHO). Conventional methods of detecting antibiotic-resistant pathogens do not assess the resistance mechanism and are often time-consuming and laborious. We have developed a magnetic digital microfluidic (MDM) platform, known as MDM Carba, for the identification of CPE by measuring their ability to hydrolyze carbapenem antibiotics. MDM Carba offers the ability to rapidly test CPE and reduce the amount of reagents used compared with conventional phenotypic testing. On the MDM Carba platform, tests are performed in droplets that function as reaction chambers, and fluidic operations are accomplished by manipulating these droplets with magnetic force. The simple droplet-based magnetic fluidic operation allows easy system automation and simplified hands-on operation. Because of the unique “power-free” operation of MDM technology, the MDM Carba platform can also be operated manually, showing great potential for point-of-care testing in resource-limited settings. We tested 27 bacterial isolates on the MDM Carba platform, and the results showed sensitivity and specificity that were comparable to those of the widely used Carba NP test. MDM Carba may shorten the overall turnaround time for CPE identification, thereby enabling more timely clinical decisions for better clinical outcomes. MDM Carba is a technological platform that can be further developed to improve diagnostics for other types of antibiotic resistance with minor modifications.

## Introduction

Infectious diseases are one of the leading causes of death, claiming over 15 million lives globally each year^1^. Managing infectious diseases is a daunting task. Fifty years after the US Surgeon General declared victory against infectious diseases^2^, the world remains in constant struggle with a series of outbreaks and pandemics. It is now well understood that the pathogens that cause infectious diseases are constantly evolving and acquiring resistance against antimicrobial agents. Antibiotics, which were once taken for granted as effective cures for infectious diseases, are losing their potency due to antibiotic resistance. The emergence of antibiotic resistance is an important crisis that could have catastrophic consequences, posing serious public health and national security threats.

Among all drug-resistant bacterial pathogens, Gram-negative bacterial pathogens are of particular concern due to increasing abundance^3–5^ and panresistance to nearly all available antibiotics^6^. One group of drug-resistant Gram-negative pathogens, known as carbapenemase-producing *Enterobacteriaceae* (CPE), is classified as a critical threat by the World Health Organization (WHO), an even higher threat level than that associated with methicillin-resistant *Staphylococcus aureus* (MRSA)^7^. The number of reported cases of CPE infections has been increasing^8,9^, and these infections are often associated with a high morbidity and mortality rate^10^. The main resistance mechanism of CPE is the production of carbapenemase—the enzyme that inactivates carbapenem—which is considered a last-line antibiotic for use against bacterial infections. Given that novel antibiotic agents, such as beta-lactam/beta-lactamase inhibitor combinations (e.g., avibactam, relebactam, and varbobactam combinations), may not be accessible or suitable^11,12^, rapid detection of CPE may enable timely clinical decisions on whether to administer last-resort but more toxic treatments, such as polymyxins, and this decision has significant clinical implications. Determining the resistance mechanisms and not merely detecting the existence of resistance may become increasingly important, as these mechanisms may affect the choice of antimicrobial therapy, and rapid results may allow more timely and appropriate therapy^13^. Conventional phenotype-based approaches for assessing the resistance mechanism, such as CPE detection based on the modified Hodge test^14,15^ or modified carbapenem inactivation method (mCIM)^16^, are time-consuming (16–24 h), and the test accuracy and specificity are suboptimal in some cases^8,17,18^. Nucleic acid-based molecular testing (e.g., PCR) for antibiotic resistance remains costly and resource-demanding and generally detects only a limited number of targets incorporated in the assay^19,20^. Many biosensing platforms have been developed for phenotypic antimicrobial susceptibility testing. However, while they can detect the presence of antibiotic resistance, they are unable to determine the resistance mechanism and hence are unsuitable for CPE detection^21–24^. Currently, there remains an unmet need for a miniaturized biosensing platform for rapid, accurate, and parallel CPE detection.

In 2012, Nordmann et al. described a rapid chromogenic assay for the detection of CPE within 2 h^19,25^, known as the Carba NP test, for which several modifications have been described since. This assay detects the resistance mechanism by measuring the activity of carbapenemase. Biomerieux offers a CPE detection kit based on the Carba NP test, which makes the assay less complex, but it still involves several manual liquid-handling procedures for each test.

We explore modifications to the Carba NP test that allow miniaturization and parallel testing of CPE by introducing magnetic digital microfluidics (MDM) technology^26–28^. By digitalizing liquids into discrete droplets, MDM accomplishes a full range of fluidic operations, such as liquid merging, mixing, dispensing, and serial dilution, upon the application of magnetic force through added magnetic particles^29–33^. Compared with conventional continuous-flow microfluidics for Carba NP^34^, MDM obviates the need for valving and pumping systems, thereby offering an easy way of handling small amounts of fluids in bioanalytical assays and a potential solution for simple automation. The use of MDM was previously demonstrated for sample-to-answer molecular testing^32,33,35–37^, immunotesting^27,30^, and broth microdilution-based antimicrobial susceptibility testing^31^. Compared with digital microfluidics based on electrowetting on dielectrics (EWOD), the droplet manipulation and system automation of MDM are less efficient, but MDM is more suitable for conducting biochemical reactions, particularly point-of-care (POC) diagnostics, due to its simple fluidic manipulation and the dual functions of magnetic particles. Recently, a 3D-printed modular MDM platform was reported for on-demand POC diagnostics^30^. These successful applications showed the suitability of MDM for in vitro diagnostic platforms^38^.

**Fig. 1:**
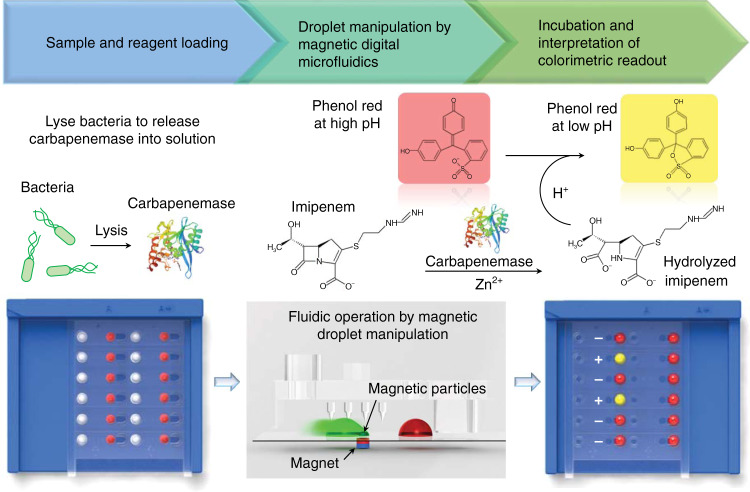
The workflow of MDM Carba. MDM Carba is a magnetic digital microfluidics-based diagnostic platform for carbapenemase-producing Enterobacteriaceae (CPE) detection

In this paper, we describe the development of an MDM-based diagnostic platform—MDM Carba—for rapid, accurate, and parallelized solutions for clinical CPE detection (Fig. 1). MDM Carba implements Carba NP^19,25,39^ to detect CPE with reduced reagent consumption by up to 90%. At the core of MDM Carba is a magnetic droplet manipulating system that utilizes a novel 3D-printed droplet manipulator and a magnet array to control 12 droplets in parallel for multiplexed detection. Droplet manipulation is automated and controlled remotely via Bluetooth using a smartphone application or a PC program. By tapping into the “power-free” operation of MDM^26^, a manual droplet manipulator was also developed to cater to potential POC applications in resource-limited settings. The MDM Carba device is described in this paper, and CPE detection using MDM Carba was demonstrated in this pilot study by testing 27 bacterial isolates and benchmarking against molecular testing and conventional Carba NP.

## Results and discussion

### System overview

The biochemical assay used to detect CPE, known as Carba NP^19^, measures the change in pH caused by the hydrolysis of imipenem by carbapenemase produced by the bacteria. The lysis buffer is colorless, while the reaction buffer is originally red. If the bacterial strain is resistant, the carbapenemase produced by the bacteria hydrolyzes imipenem and reduces the pH, causing the color of the reaction droplet to change from red to yellow or orange (Fig. 1). If the bacterial strain is susceptible, imipenem cannot be hydrolyzed, and the reaction droplet remains red. An additional control reaction is performed for each bacterial sample. The control reaction does not contain imipenem, and hence, the color of the control reaction droplet should stay red for the assay to be valid. In this study, both yellow and orange droplets were recorded as positive, and red droplets were recorded as negative.

### MDM Carba device operation

**Fig. 2:**
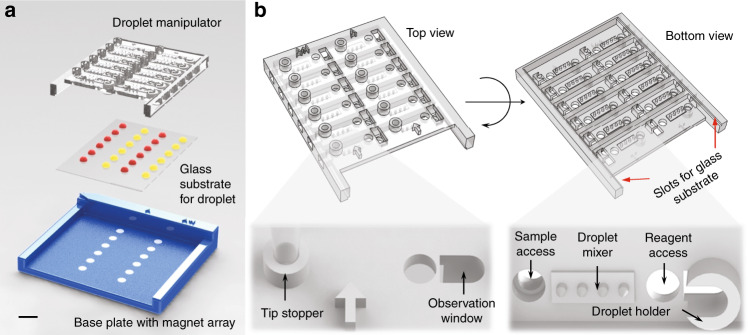
MDM Carba device. **a** Three main components of the MDM Carba device. **b** Top and bottom views of the top droplet manipulator with all the functional structures. The scale bar is 1 cm

The MDM Carba device consists of three functional parts: a base plate embedded with an array of NdFeB magnets, a Teflon-modified glass substrate, and a top droplet manipulator with assistive structures for droplet manipulation (Fig. 2a). The overall dimension of the top droplet manipulator dimension is 54 × 69 × 6.2 mm (Fig. 2b), which is designed to fit the Teflon-modified glass substrate that is 48 × 65 × 0.15 mm in size. The glass substrate is coated with Teflon AF to facilitate droplet movement. The droplet manipulator consists of 12 individual functional units to perform 12 reactions in parallel, with six on the left for sample testing and six on the right for the reaction controls. The units on the left are labeled with “A + I”, which stands for Solution A mixed with imipenem, and the ones on the right are labeled with “A” for Solution A. Each functional unit comprises one sample access port with a tip stopper, a micropillar-based passive mixer, a reagent access port, and an observation window with a droplet holder (Fig. 2b). The droplet manipulator is translucent so that the location of the droplets can be observed through the droplet manipulator. The semicircular droplet holders on the droplet manipulator are rendered hydrophilic by a polydopamine coating^27,40^.

To operate the MDM Carba device, the Teflon-modified glass substrate is slid into the slot of the droplet manipulator, and it is placed on top of the magnetic base plate in the rightmost position so that the sample and reagent access ports are aligned with the 12 neodymium magnets embedded in the base plate (Fig. 1). The distance between the droplet manipulator and the glass substrate was designed according to the height of the droplets (Table S1). The reagent solution droplets and magnetic particles are loaded onto the device using a multichannel micropipette. Ten microliters of lysis buffer (Bacterial Protein Extraction Reagent (B-PERII), Thermo Scientific) is loaded through the sample access port, followed by adding 3.5 μL of magnetic particles (MagAttract Suspension G, Qiagen) into each droplet. Then, 10 μL of Solution A (0.5% w/v phenol red solution (Sigma-Aldrich) with 10 mM zinc sulfate (Sigma-Aldrich) buffered to pH 7.8 by adding 0.1 N NaOH) is loaded via the reagent access ports into the reaction units on the right labeled with “A”. Solution A with an additional 12 mg/mL imipenem/cilastatin (Fresenius Kabi), which contains 6 mg/mL imipenem, is loaded via the reagent access ports into the reaction units on the left labeled with “A + I”. Bacterial colonies are picked by using a 200-μL pipette tip. The tip with the bacterial colony is inserted into the sample droplet through the sample access port and incubated for 5 min. After that, the device is operated by sliding the top droplet manipulator together with the glass substrate on the base plate. Finally, the colorimetric results are recorded and analyzed.

### Automated MDM Carba device

**Fig. 3:**
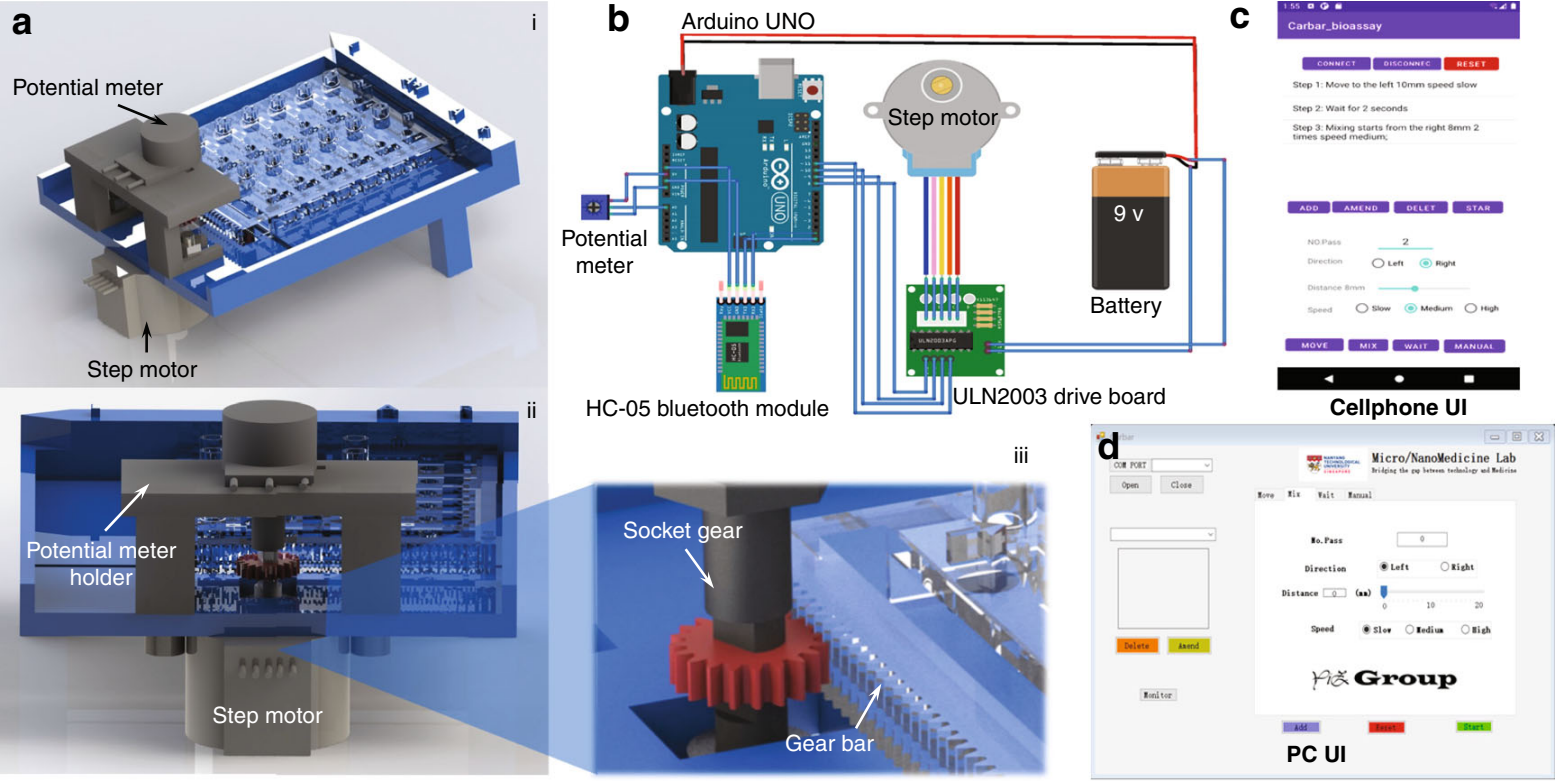
Automated MDM Carba device. **a** Schematic illustration of the automated MDM Carba device. **b** Schematic of the control circuit. **c** Cellphone user interface and **d** PC user interface

A motorized version of MDM Carba was developed for automated operation. The automated MDM Carba device consists of a modified top plate with a gear bar, a potentiometer, a step motor, and a socket gear (Fig. 3ai). The potentiometer holder and socket gear (Fig. 3aii) are designed according to the ANSI standard with a module of 0.5 and a pressure angle of 20°. A spur gear attached to a step motor is coupled to the linear gear rank of the droplet manipulator and functions as an actuator. As the spur gear rotates, the droplet manipulator moves along the base plate, and its location is sensed by the potentiometer (Fig. 3aiii).

The control mechanism consists of a potentiometer, an Arduino UNO, an HC-05 Bluetooth module, a 28byj step motor, a ULN2003 drive board, and a 9-V battery (Fig. 3b). The 28byj-48 step motor, operating in four phases and unipolar mode, is used to drive the droplet manipulator. The motor was set at 200, 400, and 600 rpm according to the speed requirements, and the corresponding moving speeds of the droplet manipulator were measured to be 3.28, 6.57, and 9.85 mm/s, respectively. A potentiometer with a resistance of 10 kΩ and an output voltage ranging from 0 to 5 V provides position feedback. It is attached to the shaft of the step motor via the socked gear. The control system is connected to a cellphone-based user interface (UI) (Fig. 3c) via Bluetooth or a PC-based user interface (Fig. 3d) via either Bluetooth or USB connection. The Arduino board communicates with the PC via a serial port. HC-05 is connected to Arduino and the computer via Bluetooth. The cellphone application and PC program allow the user to set the speed, direction, and distance of movement, the movement sequence, and the waiting interval between steps in a movement sequence.

Several commands are defined in Arduino by using the C language to allow the user to adjust and define the target locations of the droplet manipulator. The software to control the droplet manipulator in PC and cellphones was built with Visual Studio 2019 (Winform) and Android studio (Java), respectively. Three basic operations, namely, “Move”, “Mix”, and “Wait”, are defined. In “Move”, the user defines the moving distance and direction; in “Mix”, the user defines the number of passes for mixing; and in “Wait”, the user defines the duration of waiting. A detailed description of the control software is available in Fig. S2.

### MDM Carba validation

**Fig. 4:**
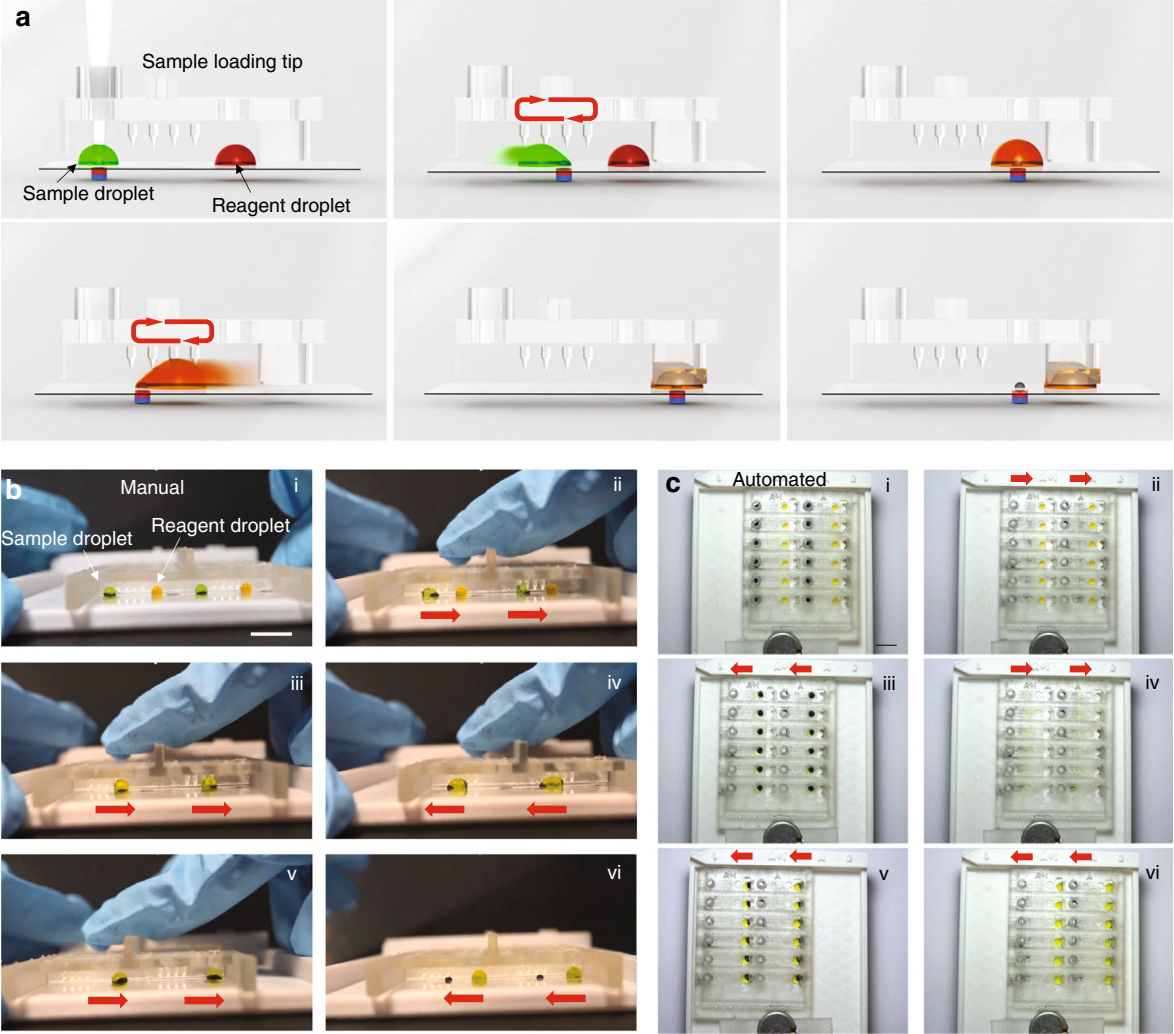
Operation of MDM Carba. **a** Schematic illustration of the operation procedures. **b** Demonstration of the operation procedures of the manual MDM Carba platform (side view). **c** Demonstration of the operation procedures of the automated MDM Carba platform (top view). All scale bars are 1 cm

To determine whether a bacterial strain is a CPE species using MDM Carba, two reactions are required. One of the reactions contains imipenem/cilastatin in the reaction buffer, and the other reaction, lacking imipenem, serves as the control. On the MDM Carba platform, a total of 12 reactions were performed in parallel to test six bacterial strains, and each bacterial strain was tested in the two reaction units in the same row. The operation procedure to detect CPE on MDM Carba is illustrated in Fig. 4. Water droplets stained with food color were used to demonstrate droplet manipulation. Before introducing bacterial samples into the MDM Carba platform, all reagents were loaded onto the glass substrate via the access ports on the droplet manipulator. The sample droplets are represented as green droplets (Fig. 4a), and the reagent droplets, either solution A or solution A with imipenem/cilastatin, are represented as yellow droplets (Fig. 4a). The volume ratio of the two droplets was maintained at 1:1. In the current design, the volume of the droplet should be greater than 8 μL; otherwise, the droplet will not be tall enough to reach the mixing pillar (Table S2). The maximal volume of each droplet is 11 μL; otherwise, the merged droplet becomes larger than 22 μL and becomes stuck easily between two mixing pillars. Droplets of 10 μL in volume were used for CPE detection in this study.

To initiate the reaction, a bacterial colony is added to the sample droplet by using a bacterial colony picker (in this case, standard 200-μL pipette tips were used). The stopper at the sample access port prevents the user from overinserting the picker and damaging the surface coating of the glass substrate (Fig. 4a). The stopper is designed in such a way that the tip of the bacteria colony picker is accurately inserted into the center of the sample droplet when the picker rests on the stopper. The position of the tip can be observed via the 6-square windows on the left side of the droplet manipulator (Fig. 2b). After resting on the stopper for 5 min, the bacterial colony picker is removed. Fig. 4b and 4c demonstrate droplet manipulation on both the manual (Video S1a side view and Video S1b top view) and automated (Video S2 top view) MDM Carba platforms.

As the droplet manipulator and glass substrate slide against the base plate either manually (Fig. 4b) or with the automated control system (Fig. 4c), the sample droplet with magnetic particles inside is anchored to the magnet array. A schematic illustration of droplet motion is shown in Fig. 4a. The red arrows indicate the movement direction of the droplets relative to the droplet manipulator. To facilitate bacterial lysis and sample mixing, the sample droplets with bacteria inside are moved back and forth under the mixer for a few passes (Figs. 4aii, bii and cii). As the droplets pass the mixing pillars, they are stretched and released repetitively by the mixing pillars, which induces efficient mixing inside the droplet. The micropillar-based passive mixer was extensively characterized in our previous study^30^. Droplets are able to achieve 90% homogeneity after moving through the mixer with only two passes. In contrast, at least 6 passes are required without the mixer (Fig. S1). Next, the sample droplets are moved to the right to merge with the reaction buffer droplets (Fig. 4aiii, biii, and ciii). The merged droplets are then moved back and forth under the mixer again to promote the mixing of the sample and reaction buffer (Figs. 4aiv, biv, and civ). Then, the merged droplets are moved toward the observation windows, where they are immobilized by the droplet holders (Figs. 4av, bv, and cv). To facilitate the colorimetric readout, the magnetic particles are removed from the droplets (Figs. 4avi, bvi, and cvi). The droplet holders at the observation windows are coated with polydopamine^27,40^, which makes them hydrophilic and capable of holding the droplets in position while the magnetic particles are extracted from the droplets. Finally, the droplets are incubated at room temperature in a humid chamber. The colorimetric readouts are recorded at 30, 45, 60, and 120 min. The overall sample-to-answer time is ~125 min, regardless of whether the operation is manual or automated (Table S4). The time could be shortened to 65 min by reducing the incubation time to 60 min without compromising the sensitivity (Table S4).

**Fig. 5:**
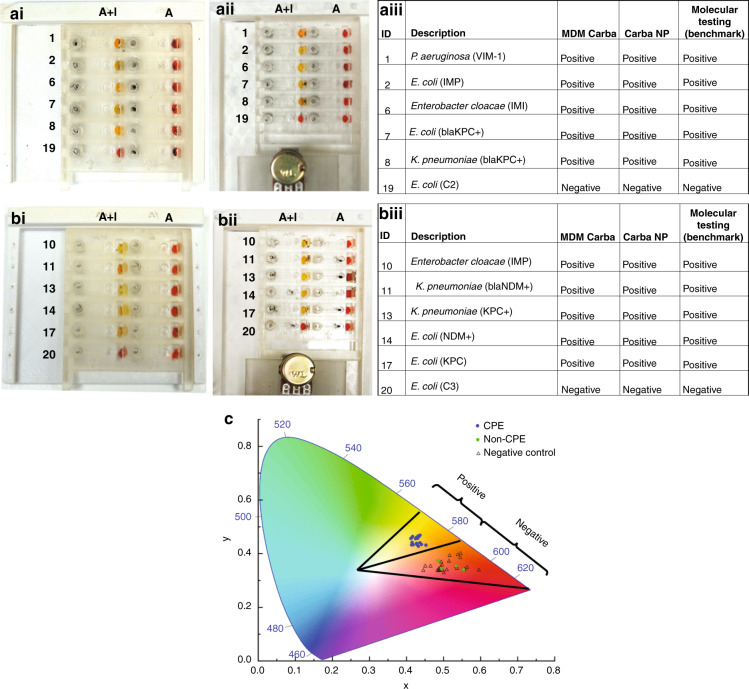
Representative testing results. Testing results on the **ai**, **bi** manual MDM Carba platform and **aii**, **bii** automated MDM Carba platform. **aiii** and **biii** Summary of the testing results in **i** and **ii**. **c** Mapping of the RGB color of droplets to the CIE 1931 color space. All scale bars are 1 cm

Two representative sets of MDM Carba tests are shown in Fig. 5. For demonstration, 5 CPE strains and 1 non-CPE strain were tested on each MDM Carba platform, and the results obtained by manual operation (Figs. 5ai and 5bi) and automated operation (Figs. 5aii and 5bii) were compared. Both manual and automated MDM Carba showed the same results. All droplets for control reactions in column “A” showed a red color, indicating that the test reactions were valid. All CPE strains caused the color of the droplets in the “A + I” column to change from red to yellow/orange (rows 1–5 in each panel) due to the hydrolysis of imipenem by carbapenemase. In contrast, the non-CPE strains did not lead to any color change, and the color of the droplets containing non-CPE strains in the “A + I” column remained red. The results obtained by MDM Carba were consistent with the results of the conventional Carba NP test and molecular testing for these two subsets of samples (Figs. 5aiii and 5biii). In conventional Carba NP, the color of the reaction is determined subjectively by the operator. We attempted to objectively determine the color of the reaction droplets by mapping their RGB values into the CIE 1931 color space (Fig. 5c). A positive region (yellow/orange) and a negative region (red) were defined in the color space. The CPE strains fell into the positive region, and the non-CPE strains fell into the negative region. The color of the control reactions was red, and hence, a test was valid only if the control reaction fell into the negative region.

We validated MDM Carba with a total of 27 clinical isolates, of which 15 were CPE strains and 12 were non-CPE strains (Table 1). Each strain was tested in triplicate, and a result was considered positive if at least 2 out of 3 replicates were positive. Both MDM Carba and conventional Carba NP exhibited 100% specificity. This is consistent with findings reported earlier with conventional Carba NP^25^. Cohen’s kappa (*κ*) was determined to statistically evaluate the degree of agreement between MDM Carba and Carba NP. The results showed *κ* = 0.851 (95% CI, 0.655–1.047), indicating good agreement between the two tests based on the Landis and Koch classification. The sensitivity was 86.7% for the conventional Carba NP test and 73.3% for MDM Carba. The 13.3% (95% CI, −9.46 to 36.2) difference in sensitivities between MDM Carba and conventional Carba NP was not statistically significant at a 5% level of significance (*p* = 0.480) based on McNemar’s test. Most isolates, except for a few strains in the VIM and OXA-48-like groups, were identified as positive at 30 min (Table S4). The bacterial isolates that yielded false negatives at 120 min also belonged to the VIM and OXA-48-like groups. This is consistent with findings from earlier studies that reported difficulty in detecting OXA-48-like carbapenemases by Carba NP^25,41^, indicating that the Carba NP test had a lower sensitivity against OXA-48-like CPE due to its weaker carbapenemase activity and occasionally due to the presence of mucoid strains. Among the isolates that gave positive results in both tests, the average time to color change was 49.8 min for MDM Carba and 41.4 min for Carba NP. The slower reaction rate and the slightly higher false-negative rate of MDM Carba were likely caused by the low concentration of the B-PERII reagent in the sample droplet. Only 10% instead of 1× B-PERII was used per reaction due to the low surface tension of the B-PERII reagent. When used at higher concentrations, B-PERII in the droplet led to a low contact angle (Table S2) and strong adhesion to the glass substrate, despite the Teflon AF coating. There are two potential solutions to this problem for future development. One solution is to change the assay to one that does not require the B-PERII reagent (e.g., Carba blue^42^), and the other solution is to coat the glass substrate with a superhydrophobic layer to reduce the adhesion of the droplet. In addition, spectrometric readouts could also be incorporated to further improve the detection sensitivity^43^.

**Table 1:** Summary of testing results

Reference isolate			MDM Carba test results	Carba NP test results
Sample ID	β-Lactamase class	Species		
CPE				
7	KPC	*E. coli* 6013499989	Positive	Positive
17	KPC	*E. coli* 7013614501	Positive	Positive
8	KPC	*K. pneumoniae* 6033440078	Positive	Positive
13	KPC	*E. pneumoniae* ATCC BAA 1705	Positive	Positive
14	NDM	*E. coli* MBRL 235	Positive	Positive
11	NDM	*K. pneumoniae* 2073318014	Positive	Positive
3	OXA-48	*K. pneumoniae* NCTC 13442	Negative	Positive
18	OXA-48	*K. pneumoniae* RB0384N1	Negative	Positive
5	OXA-232	*K. pneumoniae* 5063645495	Negative	Negative
4	VIM	*K. pneumoniae* NCTC 13439	Negative	Negative
1	VIM	*P. aeruginosa* MBRL 318	Positive	Positive
2	IMP	*E. coli* NCTC 13476	Positive	Positive
10	IMP	*Enterobacter cloacae* 5083627155	Positive	Positive
9	IMP	*S. marcescens* 6013550755	Positive	Positive
6	IMI	*Enterobacter cloacae* 6013494006	Positive	Positive
Non-CPE				
12	AmpC	*Enterobacter cloacae* ATCC BAA 1143	Negative	Negative
16	CTXm-15	*E. coli* MBRL 503	Negative	Negative
15	None	*E. coli* ATCC 25922	Negative	Negative
19	None	*E. coli* C2 (6123–141343)	Negative	Negative
20	None	*E. coli* C3 (6123–570391)	Negative	Negative
22	None	*E. coli* C6 (6123–142017)	Negative	Negative
24	None	*E. coli* C8 (6123–143336)	Negative	Negative
27	None	*E. coli* C18 (7013–614848)	Negative	Negative
21	None	*K. pneumoniae* C4 (6123–565674)	Negative	Negative
23	None	*K. pneumoniae* C7 (6123–143006)	Negative	Negative
25	None	*K. pneumoniae* C10 (6123–572315)	Negative	Negative
26	None	*K. pneumoniae* C13 (6123–145679)	Negative	Negative

A total of 27 isolates, with 15 CPE strains and 12 non-CPE strains, were tested with MDM Carba, and the results were compared with those of conventional Carba NP

*KPCK. pneumoniae* carbapenemase, *NDM* New Delhi metallolactamase, *OXA-48* oxacillinase-48, *OXA-232* oxacillinase-232, *VIM* Verona-integron-encoded metallolactamase, *IMP* imipenem, *IMI* imipenem-hydrolysing lactamase, *AmpC* ampicillin C, *CTX-M-15* cefotaximase-Munich 15

Conventional Carba NP is tedious to perform and analyzes only one clinical isolate at a time, which is time-consuming and labor-demanding in diagnostic settings wherein a large number of clinical isolates need to be tested. With MDM Carba, we can manipulate an array of droplets to test multiple bacterial samples in parallel with automated operation. In addition, MDM Carba reduces reagent consumption and hence the cost by up to 90%. Furthermore, the same magnetic digital microfluidic platform could be applied to phenotypical testing of other antibiotic resistance mechanisms (e.g., extended-spectrum beta-lactamases, aminoglycoside, and polymyxin resistance) with minor modifications. In clinical laboratories, MDM Carba could be operated in an automated mode. All the users need to do is load the sample, and the testing results will be made available with the click of a button, significantly reducing hands-on operations. Several conventional microfluidic platforms show great potential for rapid antimicrobial testing (AST) by analyzing pathogens at the single-cell level^44–46^. These platforms indicate a possible direction for the future development of AST, but they are relatively more resource-demanding and better suited for research in centralized facilities. Another major advantage of magnetic digital microfluidics is the “power-free” manual operation^26^. Unlike conventional microfluidic systems, magnetic digital microfluidics does not require any pumping or valving equipment for operation, and hence, it is well suited for point-of-care testing in resource-limited settings. Indeed, the manual MDM Carba platform, with its simple method of liquid manipulation, shows great promise for potential POC testing of antibiotic resistance.

In conclusion, antibiotic resistance is on the rise and has become a global concern. Rapid identification of antibiotic resistance and the underlying mechanism could guide timely clinical decisions and lead to better clinical outcomes. We have developed a magnetic digital microfluidic platform called MDM Carba for detection of CPE—a type of Gram-negative bacteria that are resistant to carbapenems. MDM Carba has shown comparable sensitivity and specificity to conventional Carba NP. In addition, MDM Carba offers many advantages. It allows parallel sample processing with reduced reagent consumption, easy fluidic manipulation, and simple hands-on operation. Not only can MDM Carba be easily automated, but the same design can also be operated manually for applications in low-resource settings due to the unique advantage of MDM. Because of its “power-free” operation and compact size, MDM Carba is easily portable, which makes the device user-friendly and well-suited for potential point-of-care testing. The current study is not without limitations. The purpose of this work was to demonstrate a proof of concept for MDM-based CPE detection. The sample size was hence relatively small, with only a total of 27 bacterial strains, and the tests were not blinded. In future work, we will further optimize MDM Carba and test a large number of samples in a double-blinded manner. The platform can be scaled up by increasing the number of functional units per device and running multiple devices in parallel to enhance the testing throughput in future development.

## Materials and methods

### Bacterial samples

Bacterial isolates that were confirmed to be CPE by molecular testing were provided by the Infectious Disease Research Laboratory (IDRL) at the National Centre for Infectious Diseases (NCID), Singapore, and the Mayo Clinic (Rochester, MN). Non-CPE control strains from ATCC and NCTC were also used. There were 16 CPE bacterial isolates (positive samples), including isolates from the KPC (4), NDM (2), OXA-48 (2), OXA-232 (1), VIM (2), IMP (3), and IMI (1) classes, and 11 non-CPE bacterial isolates (Table S3). All the isolates were transported and stored in Microbank vials (Pro-Lab Diagnostics, ON, Canada) at −80 °C.

Before inoculation, all the bacterial isolates were subcultured twice from the Microbank vials stored at −80 °C with a 1-μL inoculating loop and plated on 5% TSA sheep blood agar plates (Thermo Fisher Scientific, MA, USA). The plates were incubated at 37 °C in ambient air overnight and were ready for inoculation after at least two subcultures. Each isolate was tested in triplicate by both MDM Carba and conventional Carba NP.

### Device fabrication

The droplet manipulator was designed using Solidworks (Dassault Systèmes) and printed by using a stereolithography (SLA) 3D printer (Form 2, Formlabs, MA, USA) with rigid clear resin. After postprinting processing, the bottom surface of the droplet manipulator was dip-coated with 1% Teflon AF solution (Dupont^TM^,Wilminton, DE) and dried at room temperature overnight. The droplet holder was coated with polydopamine. Polydopamine coating was performed by dipping the droplet holder in 5 mg/mL dopamine-HCL solution (Sigma-Aldrich, MU, Germany) dissolved in 10 mM Tris at pH 8.8 (1^st^ base, Axil Scientific, Singapore) for 3 h in a humidity-controlled chamber. The base plate was surrounded by three walls and had an overall dimension of 85 × 75.5 × 14 mm. It was printed by using a fused deposition modeling (FDM) 3D printer (Dreamer, Flashforge 3D Technology, Zhejiang, China) with white acrylonitrile butadiene styrene (ABS). Twelve 4 × 1-mm (diameter x height) cylindrical NdFeB magnets with an average magnetic strength of 210 mT were embedded in the base plate. Before starting the experiment, the glass substrate (Thermo Fisher, MA, USA) was spin-coated with 1% Teflon AF solution (Dupont^TM^, DE, USA) for 40 s at 500 rpm and incubated at 120 °C on a hot plate for 3 min. The potentiometer holder and the gears of the automated MDM Carba device were fabricated by using a Nobel 1.0 A stereolithography (SLA) printer with rigid clear resin. After each use, the droplet manipulator was cleaned by soaking in ethanol, rinsing with water, drying, and sterilizing by ultraviolet light for 10 min before reuse. The glass substrate was discarded, and the base plate could be directly reused because it was not in contact with the bacterial samples.

### Carba NP test

Conventional Carba NP was performed by using a modified protocol described by Vasoo et al.^25^, who performed the testing in a pair of microcentrifuge tubes. Each tube contained 100 μL of 20 mM bacterial protein extraction reagent (B-PERII, Thermo Scientific, MA, USA). Next, a 1-μL loopful of the bacterial sample was inoculated into the reagent and vortexed for 5 s. This method used the cultures directly instead of using the supernatant of lysed cells, thereby obviating the need for a centrifuge. One hundred microliters of solution A (0.5% w/v phenol red solution (Sigma-Aldrich, MU, Germany) with 10 mM zinc sulfate (Sigma-Aldrich, MU, Germany) buffered to pH 7.8 by adding 0.1 N NaOH) was added to the first tube, and 100 μL of solution A containing 12 mg/mL imipenem/cilastatin (Fresenius Kabi, Bad Homburg, Germany), which contained 6 mg/mL imipenem, was added to the second tube. The protocol used in this study was modified by downscaling the volume of each reagent to 10 μL (90% reduction in volume) and performing the reaction in droplets on the MDM Carba platform. The incubation was performed at room temperature instead of 37 °C.

### Data analysis

The phenotypic results were determined visually or by analyzing the RGB values of the droplets with ImageJ (NIH). The RGB values were mapped into XYZ color space and normalized into *xy*-coordinates in a two-dimensional chromaticity diagram (CIE 1931 color space diagram).

The statistical analysis was performed by using SPSS statistical software. To compare the efficacy of MDM Carba with that of Carba NP, Cohen’s kappa (*κ*) and McNemar’s tests were run to statistically evaluate the degree of agreement between the results obtained by MDM Carba and conventional Carba NP.

## Supplementary information

Supplementary Information

Video S1a

Video S1b

Video S2
