# Supplementary material for: A 3D-printed magnetic digital microfluidic diagnostic platform for rapid colorimetric sensing of carbapenemase-producing Enterobacteriaceae
Source: Microsyst Nanoeng. 2021 Jun 12;7:47. doi: 10.1038/s41378-021-00276-9 (PMC8433351; doi:10.1038/s41378-021-00276-9)
Supplement: Supplementary file 1 — Supplementary Information [file 41378_2021_276_MOESM1_ESM.docx]

**Supplementary Information: A 3D-Printed Magnetic Digital Microfluidic Diagnostic Platform for Rapid Colorimetric Sensing of Carbapenemase-Producing *Enterobacteriaceae***

Kanitthamniyom Pojchanun^1^, Pei Yun Hon^2^, Aiwu Zhou^1^, Mohammad Yazid Abdad^2^, Zhi Yun Leow^3^, Nurhidayah Binte Mohamed Yazid^2^, Vanessa Lim Wei Xun^2^, Shawn Vasoo^2^* and Yi Zhang^1^*

^1^Singapore Centre for 3D Printing, School of Mechanical and Aerospace Engineering, Nanyang Technological University, Singapore, Singapore

^2^ National Center for Infectious Disease, Tan Tock Seng Hospital, Singapore, Singapore

^3^Lee Kong Chian School of Medicine, Nanyang Technological University, Singapore, Singapore

Correspondence should be addressed to Yi Zhang (yi_zhang@ntu.edu.sg) and Shawn Vasoo (shawn_vasoo@ncid.sg)

**Supplementary Information 1:** Droplet characterization

a)

| **Solution A** | | | |
| --- | --- | --- | --- |
| **Volume (μL)** | **Contact angle (°)** | **Length (mm)** | **Height (mm)** |
| **10.0** | 116.05 | 2.72 | 1.46 |
| **9.5** | 108.15 | 2.75 | 1.35 |
| **9.0** | 105.47 | 2.74 | 1.30 |
| **8.5** | 115.21 | 2.60 | 1.40 |
| **8.0** | 114.29 | 2.55 | 1.36 |

**b)**

| **10 μL BPERII** | | | |
| --- | --- | --- | --- |
| **%** | **Contact angle (°)** | **Length (mm)** | **Height (mm)** |
| **100** | 66.22 | 3.83 | 0.99 |
| **90** | 62.35 | 3.83 | 0.93 |
| **80** | 65.96 | 3.91 | 1.02 |
| **70** | 64.85 | 3.85 | 0.99 |
| **60** | 65.67 | 3.91 | 1.01 |
| **50** | 65.59 | 3.83 | 0.98 |
| **40** | 63.10 | 3.85 | 0.95 |
| **30** | 62.91 | 3.79 | 0.94 |
| **20** | 65.24 | 3.87 | 0.99 |
| **10** | 64.71 | 3.90 | 0.99 |
| **5** | 92.65 | 3.70 | 1.37 |

**Supplementary Tab. S1** The droplet characterization on contact angle, length, and height of droplet on Teflon coated glass coverslip. a) The different volumes of solution A droplet. b) The different concentrations of 10 ul of lysis buffer.

|  |  | **Speed** | | |
| --- | --- | --- | --- | --- |
| **10% BPERII (μL)** | **MP volume (μL)** | **low** | **medium** | **high** |
| **9** | **2** | F | P | P |
|  | **2.5** | P | P | P |
|  | **3** | P | P | P |
|  | **3.5** | P | P | P |
|  | **4** | P | P | P |
| **9.5** | **2** | P | P | P |
|  | **2.5** | P | P | P |
|  | **3** | P | P | P |
|  | **3.5** | P | P | P |
|  | **4** | P | P | P |
| **10** | **2** | P | P | P |
|  | **2.5** | P | P | P |
|  | **3** | P | P | P |
|  | **3.5** | P | P | P |
|  | **4** | P | P | P |
| **18** | **2** | F | F | F |
|  | **2.5** | P | P | P |
|  | **3** | P | P | P |
|  | **3.5** | P | P | P |
|  | **4** | P | P | P |
| **19** | **2** | F | F | F |
|  | **2.5** | P | P | P |
|  | **3** | P | P | P |
|  | **3.5** | P | P | P |
|  | **4** | P | F | P |
| **20** | **2** | F | F | F |
|  | **2.5** | P | P | P |
|  | **3** | P | P | P |
|  | **3.5** | P | P | P |
|  | **4** | P | F | P |
| **22** | **2** | F | F | F |
|  | **2.5** | P | P | F |

**Supplementary Tab. S2** The mixing ability of 10% BPERII droplet in different volumes with different volumes of magnetic particles (MP) in three-speed settings (P = pass, F = fail).

**Supplementary Information 2: Bacterial isolates**

| **Sample ID** | **Bacteria isolate species** | **𝝱-lactamase class** |
| --- | --- | --- |
| 1 | *Pseudomonas aeruginosa* MBRL | VIM-1 |
| 2 | *Escherichia coli* NCTC 13476 | IMP |
| 3 | *Klebsiella pneumoniae* NCTC 13442 | OXA-48 |
| 4 | *Klebsiella pneumoniae* NCTC 13439 | ViM-1 |
| 5 | *Klebsiella pneumoniae* 5063645495 | OXA-232 |
| 6 | *Enterobacter clocae* complex 6013494006 | IMI |
| 7 | *Escherichia coli* 6013499989 | blaKPC+ |
| 8 | *Klebsiella pneumoniae* 6033440078 | blkKPC+ |
| 9 | *Serratia marscecens* 6013550755 | IMP |
| 10 | *Enterobacter clocae* complex 5083627155(1) | IMP |
| 11 | *Klebsiella pneumoniae* 2073318014 | blkNDM+ |
| 12 | *Enterobacter clocae* ATCC BAA-1143 | AmpC |
| 13 | *Klebsiella pneumoniae* ATCC BAA-1705 | KPC+ |
| 14 | *Escherichia coli* MBRL 235 | NDM+ |
| 15 | *Escherichia coli* ATCC25922 |  |
| 16 | *Escherichia coli* MBRL 503 | CTXm-15 |
| 17 | *Escherichia coli* 7013-614501 | KPC |
| 18 | *Klebsiella pneumoniae* complex RB0384N1 | OXA-48 |
| 19 | *Escherichia coli* - C2 (6123-141343) |  |
| 20 | *Escherichia coli* - C3 (6123-570391) |  |
| 21 | *Klebsiella pneumoniae* - C4 (6123-565674) |  |
| 22 | *Escherichia coli* - C6 (6123-142017) |  |
| 23 | *Klebsiella pneumoniae* - C7 (6123-143006) |  |
| 24 | *Escherichia coli* - C8 (6123-143336) |  |
| 25 | *Klebsiella pneumoniae* - C10 (6123-572315) |  |
| 26 | *Klebsiella pneumoniae* - C13 (6123-145679) |  |
| 27 | *Escherichia coli* - C18 (7013-614848) |  |

**Table S3** Bacteria isolate sample collected from Infectious Disease Research Laboratory (IDRL), National Centre for Infectious Diseases (NCID) Singapore.

**Tab. S4** Summary of testing results. A total of 27 isolates, with 15 CPE strains and 12 non-CPE strains, are tested and compared with conventional Carba NP. Results are read at 30 mins, 45 mins, 60 mins and 120 mins.

| **Reference isolate** | | | | **MDM-Carba test results** | | | |  | **Carba NP test results** | | | |  |
| --- | --- | --- | --- | --- | --- | --- | --- | --- | --- | --- | --- | --- | --- |
| **Sample ID** | **β-lactamase class** | **Species** | **30 mins** | | **45 mins** | **60 mins** | **120 mins** |  | **30 mins** | **45 mins** | **60 mins** | **120 mins** |  |
| **CPE** | | | | | | | | | | | | |  |
| 7 | KPC | *E.coli* 6013499989 | Positive (2) Negative (1) | | Positive (2) Negative (1) | Positive (3) | Positive (3) |  | Positive (3) | Positive (3) | Positive (3) | Positive (3) |  |
| 17 | KPC | *E.coli* 7013614501 | Positive (3) | | Positive (3) | Positive (3) | Positive (3) |  | Positive (3) | Positive (3) | Positive (3) | Positive (3) |  |
| 8 | KPC | *K. pneumoniae* 6033440078 | Positive (3) | | Positive (3) | Positive (3) | Positive (3) |  | Positive (3) | Positive (3) | Positive (3) | Positive (3) |  |
| 13 | KPC | *E. pneunomiae* ATCC BAA 1705 | Positive (3) | | Positive (3) | Positive (3) | Positive (3) |  | Positive (3) | Positive (3) | Positive (3) | Positive (3) |  |
| 14 | NDM | *E. coli* MBRL 235 | Positive (3) | | Positive (3) | Positive (3) | Positive (3) |  | Positive (3) | Positive (3) | Positive (3) | Positive (3) |  |
| 11 | NDM | *K. pneumoniae* 2073318014 | Positive (1) Negative (2) | | Positive (1) Negative (2) | Positive (3) | Positive (3) |  | Positive (2) Negative (1) | Positive (2) Negative (1) | Positive (3) | Positive (3) |  |
| 3 | OXA-48 | *K. pneumoniae* NCTC 13442 | Negative (3) | | Negative (3) | Negative (3) | Negative (3) |  | Negative (3) | Positive (2) Negative (1) | Positive (2) Negative (1) | Positive (2) Negative (1) |  |
| 18 | OXA-48 | *K. pneumoniae* RB0384N1 | Negative (3) | | Negative (3) | Negative (3) | Negative (3) |  | Negative (3) | Negative (3) | Negative (3) | Positive (3) |  |
| 5 | OXA-232 | *K. pneumoniae* 5063645495 | Negative (3) | | Negative (3) | Negative (3) | Negative (3) |  | Negative (3) | Negative (3) | Negative (3) | Negative (3) |  |
| 4 | VIM | *K. pneumoniae* NCTC 13439 | Negative (3) | | Negative (3) | Negative (3) | Negative (3) |  | Negative (3) | Negative (3) | Negative (3) | Negative (3) |  |
| 1 | VIM | *P. aeruginosa* MBRL 318 | Negative (3) | | Negative (3) | Positive (2)  Negative (1) | Positive (2) Negative (1) |  | Negative (3) | Negative (3) | Positive (3) | Positive (3) |  |
| 2 | IMP | *E.coli* NCTC 13476 | Positive (2) Negative (1) | | Positive (2) Negative (1) | Positive (2) Negative (1) | Positive (3) |  | Positive (2) Negative (1) | Positive (2) Negative (1) | Positive (2) Negative (1) | Positive (3) |  |
| 10 | IMP | *Enterobacter cloacae* 5083627155 | Positive (2) Negative (1) | | Positive (2) Negative (1) | Positive (2) Negative (1) | Positive (3) |  | Positive (3) | Positive (3) | Positive (3) | Positive (3) |  |
| 9 | IMP | *S. marscecens* 6013550755 | Positive (3) | | Positive (3) | Positive (3) | Positive (3) |  | Positive (3) | Positive (3) | Positive (3) | Positive (3) |  |
| 6 | IMI | *Enterobacter cloacae* 6013494006 | Positive (3) | | Positive (3) | Positive (3) | Positive (3) |  | Negative (3) | Positive (3) | Positive (3) | Positive (3) |  |
|  |  |  |  | |  |  |  |  |  |  |  |  |  |
| **Non-CPE** | | | | | | | | | | | | |  |
| 12 | AmpC | *Enterobacter cloacae* ATCC BAA 1143 | Negative (3) | | Negative (3) | Negative (3) | Negative (3) |  | Negative (3) | Negative (3) | Negative (3) | Negative (3) |  |
| 16 | CTXm-15 | *E. coli* MBRL 503 | Negative (3) | | Negative (3) | Negative (3) | Negative (3) |  | Negative (3) | Negative (3) | Negative (3) | Negative (3) |  |
| 15 | None | *E.coli* ATCC 25922 | Negative (3) | | Negative (3) | Negative (3) | Negative (3) |  | Negative (3) | Negative (3) | Negative (3) | Negative (3) |  |
| 19 | None | *E.coli* C2 (6123-141343) | Negative (3) | | Negative (3) | Negative (3) | Negative (3) |  | Negative (3) | Negative (3) | Negative (3) | Negative (3) |  |
| 20 | None | *E.coli* C3 (6123-570391) | Negative (3) | | Negative (3) | Negative (3) | Negative (3) |  | Negative (3) | Negative (3) | Negative (3) | Negative (3) |  |
| 22 | None | *E.coli* C6 (6123-142017) | Negative (3) | | Negative (3) | Negative (3) | Negative (3) |  | Negative (3) | Negative (3) | Negative (3) | Negative (3) |  |
| 24 | None | *E.coli* C8 (6123-143336) | Negative (3) | | Negative (3) | Negative (3) | Negative (3) |  | Negative (3) | Negative (3) | Negative (3) | Negative (3) |  |
| 27 | None | *E.coli* C18 (7013-614848) | Negative (3) | | Negative (3) | Negative (3) | Negative (3) |  | Negative (3) | Negative (3) | Negative (3) | Negative (3) |  |
| 21 | None | *K. pneumoniae* C4 (6123-565674) | Negative (3) | | Negative (3) | Negative (3) | Negative (3) |  | Negative (3) | Negative (3) | Negative (3) | Negative (3) |  |
| 23 | None | *K. pneumoniae* C7 (6123-143006) | Negative (3) | | Negative (3) | Negative (3) | Negative (3) |  | Negative (3) | Negative (3) | Negative (3) | Negative (3) |  |
| 25 | None | *K. pneumoniae* C10 (6123-572315) | Negative (3) | | Negative (3) | Negative (3) | Negative (3) |  | Negative (3) | Negative (3) | Negative (3) | Negative (3) |  |
| 26 | None | *K. pneumoniae* C13 (6123-145679) | Negative (3) | | Negative (3) | Negative (3) | Negative (3) |  | Negative (3) | Negative (3) | Negative (3) | Negative (3) |  |
|  |  |  |  | |  |  |  |  |  |  |  |  |  |
| * KPC, K.pneumoniae carbapenemase; NDM, New Delhi metallo-lactamase; OXA-48, oxacillinase-48; OXA-232, oxacillinase-232; VIM, Verona-integron-encoded metallo-lactamase; IMP, imipenem; IMI, imipenem-hydrolysing-lactamase; AmpC, ampicillin C; CTX-M-15, cefotaximase-munich 15 | | | | | | | | | | | | |  |

**Supplementary Information 3:** Micropillar-based mixing

**
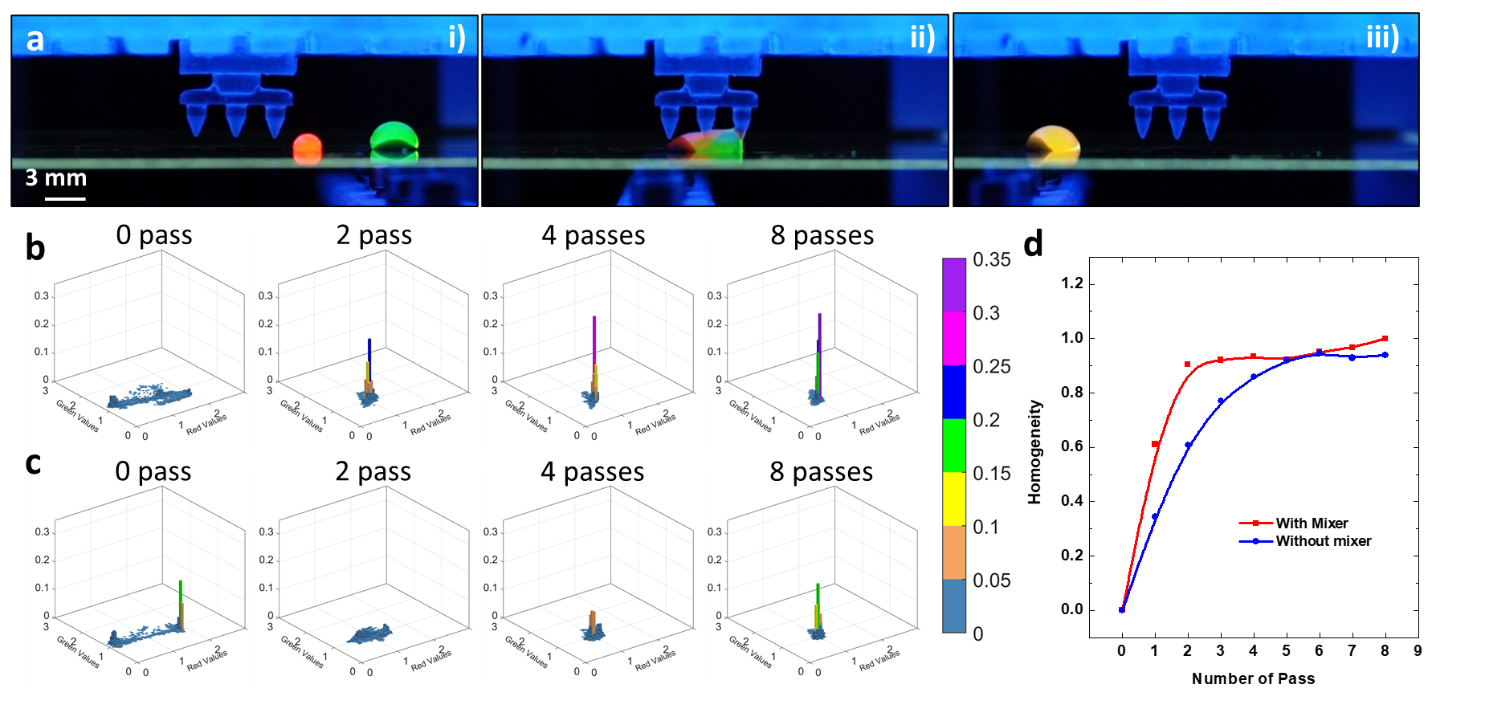
**

**Fig. S1** Droplet mixing with the mixing module. a Droplet behavior as it goes through the mixing module. b, c 2D histogram of droplet pixels in red–green color space. A single sharp peak indicates a high mixing homogeneity. b with mixer, c without mixer. d Mixing homogeneity vs number of pass. Reproduced from Ref. 30, *Microsystems & Nanoengineering*, 2020, 6, 48 with the permission from Spring Nature under the Creative Common License.

**Supplementary Information 4:** User interface of automated MDM Carba

1.
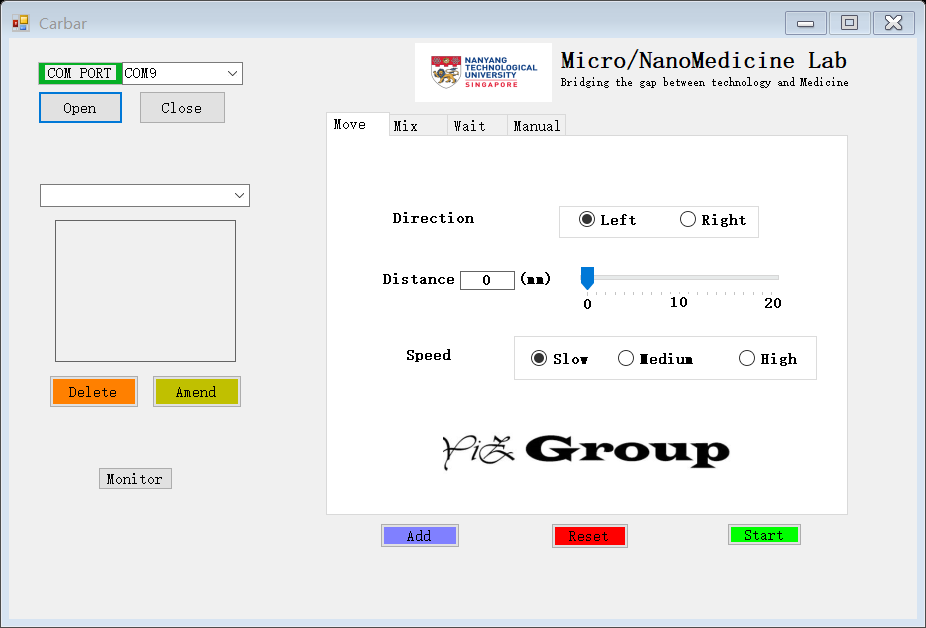
 **b)**
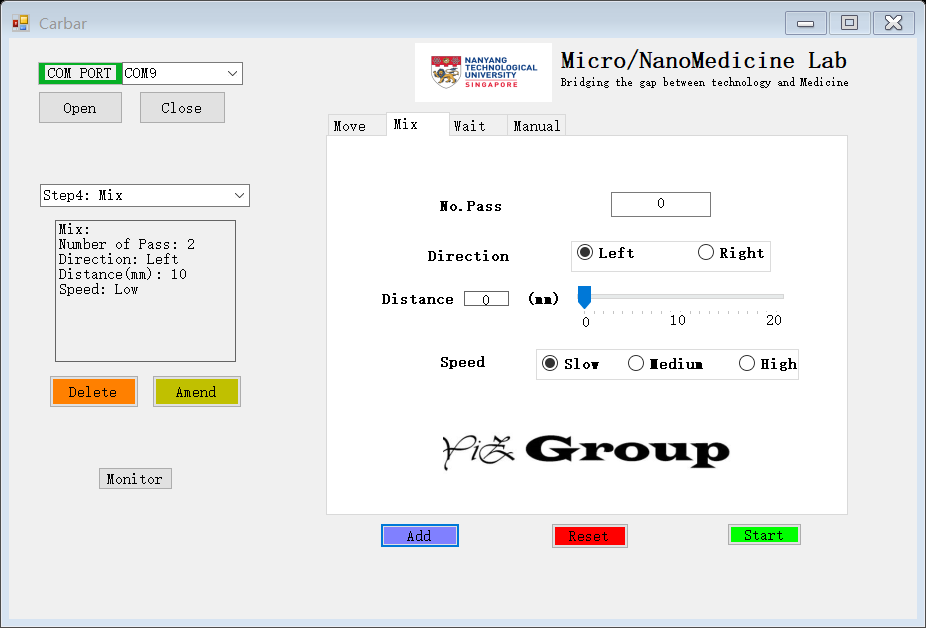

2.
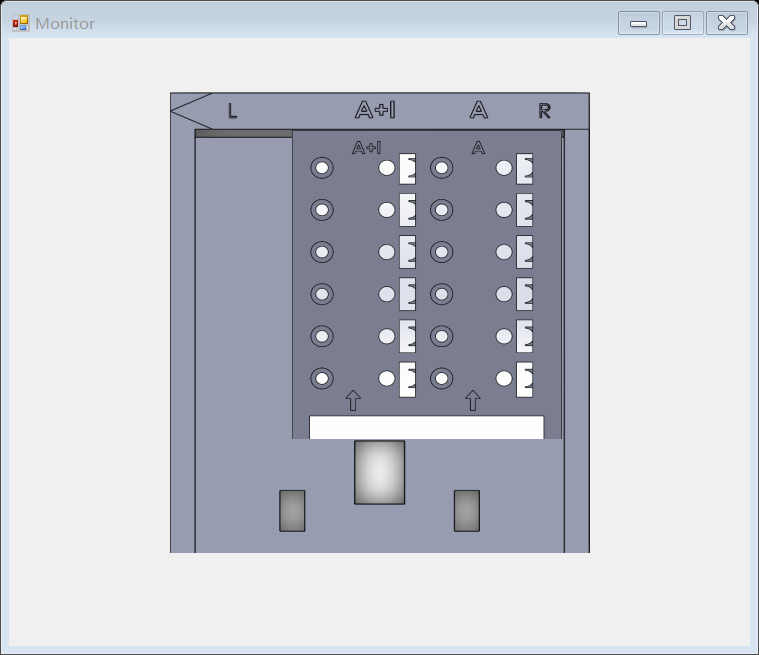
 **d)**
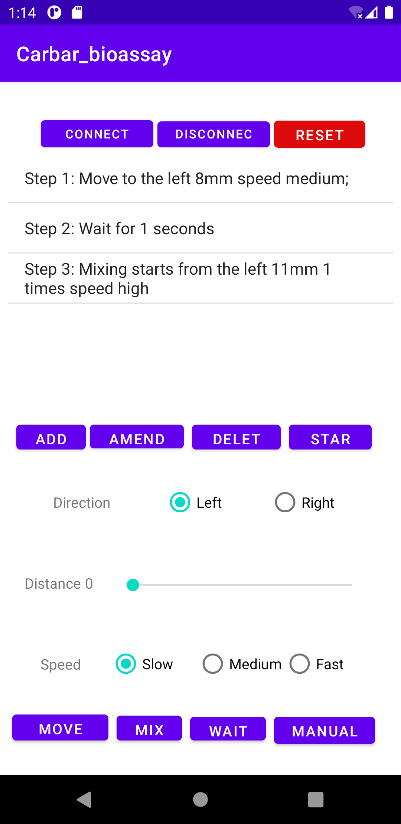


**Fig. S2** a) PC user interface, b) procedure panel, c) real-time position panel, d) cell phone user interface.

To operate the windows UI, the PC needs to be paired with via either Bluetooth or USB connections to the MDM Carba device. When the connection is established, the comport of the connected device appears in the drop-down list. The operation procedures are as follows:

1. Click “COM PORT” on the top left of the UI and choose the designated device. Then click the “open” button to start the serial communication service between the PC and device. If the service is successfully launched, the label “COM PORT” turns green.
2. Different bioassays need different procedures. The moving sequence is programmed in the procedure panel. After setting all necessary parameters in the control panel on the left of the interface, clicking the “Add” button in the procedure panel stores all the information in one step. The information of the added step appears in the information box in the procedure panel.
3. To delete a step, choose the step in the drop-down list above the information box and click the “delete” button.
4. To amend a step, choose the step in the drop-down list above the information box, change the parameters in the control panel, and click the “Amend” button.
5. When all the steps are added, click the “start” button to start the program. The “reset” button can clear all the added steps and make the chip return to the designated origin.
6. A monitor window is also provided to track the position of the chip in real-time by clicking the “Monitor” button.

To operate the cellphone Android UI, the phone needs to be paired to the MDM Carba device via Bluetooth. The connection is established by clicking the “connect” button on the top left of the UI. A pop-up window with a "Connected" message appears at the bottom of the screen once the connection is successfully established. Each step could be programmed in the same way as the programming in the windows UI.
